# Reallocating sedentary time to physical activity: effects on fatigue and quality of life in patients with breast cancer in the Phys-Can project

**DOI:** 10.1007/s00520-023-07614-9

**Published:** 2023-02-04

**Authors:** Anne-Sophie Mazzoni, Emelie Strandberg, Sussanne Börjeson, Katarina Sjövall, Sveinung Berntsen, Ingrid Demmelmaier, Karin Nordin

**Affiliations:** 1 Department of Public Health and Caring Sciences, Uppsala University, Uppsala, Sweden; 2 Department of Oncology and Department of Health, Medicine and Caring Sciences, Linköping University, Linköping, Sweden; 3 Faculty of Health Sciences, Kristianstad University, Kristianstad, Sweden; 4 Department of Sport Science and Physical Education, University of Agder, Kristiansand, Norway

**Keywords:** Fatigue, Quality of life, Breast cancer, Isotemporal substitution, Physical activity, Sedentary behaviour

## Abstract

**Purpose:**

We aimed to investigate the effects of reallocating sedentary time to an equal amount of light (LPA) or moderate-to-vigorous intensity physical activity (MVPA) on cancer-related fatigue and health-related quality of life (HRQoL) in patients with breast cancer. We also aimed to determine the daily amount of sedentary time needed to be reallocated to LPA or MVPA to produce minimal clinically important changes in these outcomes.

**Methods:**

Pooled baseline data from three studies were used, including women with breast cancer who participated in the Phys-Can project. Fatigue was assessed with the Multidimensional Fatigue Inventory questionnaire (MFI; five dimensions, 4–20 scale) and HRQoL with the European Organisation for Research and Treatment of Cancer quality of life questionnaire (EORTC QLQ-C30; 0–100 scale). Sedentary time and physical activity were measured with accelerometry. Isotemporal substitution modelling was used for the analyses.

**Results:**

Overall, 436 participants (mean age 56 years, fatigue 11 [MFI], HRQoL 66 [EORTC QLQ-C30], LPA 254 min/day, MVPA 71 min/day) were included. Fatigue significantly decreased in two MFI dimensions when reallocating 30 min/day of sedentary time to LPA: reduced motivation and reduced activity (*β* =  − 0.21). Fatigue significantly decreased in three MFI dimensions when reallocating 30 min/day of sedentary time to MVPA: general fatigue (*β* =  − 0.34), physical fatigue (*β* =  − 0.47) and reduced activity (*β* =  − 0.48). To produce minimal clinically important changes in fatigue (− 2 points on MFI), the amount of sedentary time needed to be reallocated to LPA was ≈290 min/day and to MVPA was ≥ 125 min/day. No significant effects were observed on HRQoL when reallocating sedentary time to LPA or MVPA.

**Conclusions:**

Our results suggest that reallocating sedentary time to LPA or MVPA has beneficial effects on cancer-related fatigue in patients with breast cancer, with MVPA having the greatest impact. In relatively healthy and physically active breast cancer populations, a large amount of time reallocation is needed to produce clinically important changes. Future studies are warranted to evaluate such effects in broader cancer populations.

**Trial registration:** NCT02473003 (10/10/2014) and NCT04586517 (14/10/2020).

## Background

Cancer-related fatigue, characterised by an excessive and persistent physical, cognitive and/or emotional exhaustion, is identified by patients as one of the most distressing symptoms associated with cancer and its treatment [1]. It is also one of the most frequent symptoms in patients with breast cancer, affecting approximately 60–90% of patients during and following treatment [2], and having a strong negative impact on health-related quality of life (HRQoL) [3]. Despite the well-documented benefits of moderate-to-vigorous intensity physical activity (MVPA) for improving cancer-related fatigue and HRQoL [4], patients with breast cancer tend to reduce their physical activity level after being diagnosed [5]. Whilst the majority do not meet the recommended physical activity guidelines (i.e. 150 min of MVPA) [6, 7], light-intensity physical activity (LPA) could be suggested as an alternative as exercising at this intensity has also been shown to improve health-related outcomes amongst patients with breast cancer [8–10]. In addition, performing LPA may be more feasible than MVPA for an inactive cancer population, particularly amongst those who are not able to engage in sufficient amounts of MVPA due to, e.g. treatment side effects and/or physical limitations. Moreover, patients with breast cancer spend a large amount of time sedentary [5, 7]. Sedentary time (i.e. any waking behaviour characterised by a low-energy expenditure whilst in a sitting, reclining or lying posture [11]) has emerged as a risk factor for poor health and reducing this behaviour may improve health outcomes in patients with cancer [12]. Taken all together, there is an urgent need to understand the replacing effects of sedentary time with different intensity of physical activity on health outcomes to find more appropriate physical activity prescriptions accessible for patients who are currently not meeting physical activity guidelines. For example, replacing sedentary time with LPA or MVPA could be a promising target for interventions to improve health in this population.

Isotemporal substitution modelling has been proposed as a method using cross-sectional data for analysing the effects of substituting time in one activity for another [13]. To date, a small number of studies have used this approach to explore the associations between sedentary time reallocation and different health outcomes in patients with breast cancer [14–16]. In those studies HRQoL/depression [14], body mass index (BMI) [15] and cognitive impairment [16] were examined and improvements in all the outcomes were reported when reallocating 30 min of sedentary time to MVPA. However, most of those studies had small samples [15, 16]. Furthermore, only one study has examined the effects of sedentary time reallocation on HRQoL in patients with breast cancer but the results did not reach clinically meaningful thresholds [14]. Moreover, no study has previously investigated such effects with focus on cancer-related fatigue, highlighting a need for further research. Further research is also needed to identify the thresholds of reallocation at which these effects are clinically meaningful. Such investigations will provide useful and complementary information on how to optimise exercise recommendations and thereby future exercise interventions aiming to improve cancer-related fatigue and HRQoL in patients with breast cancer.

In the present study, we aimed to investigate the effects of reallocating sedentary time to an equal amount of LPA or MVPA on cancer-related fatigue and HRQoL in patients with breast cancer, using isotemporal substitution modelling. We also aimed to determine the daily amount of sedentary time needed to be reallocated to LPA or MVPA to produce minimal clinically important changes in cancer-related fatigue and HRQoL. We hypothesised that reallocating sedentary time to an equal amount of LPA or MVPA would be beneficial for patients with breast cancer in terms of lower cancer-related fatigue and higher HRQoL. We also hypothesised that sedentary time reallocation to MVPA would have a greater effect on cancer-related fatigue and HRQoL than LPA.

## Methods

### Participants and settings

In the present study, we used baseline data from the Physical training and Cancer (Phys-Can) project. This project consists of three studies: a randomised controlled trial (RCT) with a 2 × 2 factorial design (study 1) (NCT02473003) [17], an observational study (study 2) and a two-armed RCT (study 3) (NCT04586517) [18]. Full details of individual study designs, recruitment processes and inclusion criteria have been previously described [17, 18]. Briefly, eligible patients were recruited at university hospitals in Sweden between September 2014 and September 2021. Patients were aged 18 years or older, newly diagnosed with cancer (breast, colorectal or prostate cancer in studies 1 and 2, only breast cancer in study 3) and scheduled to undergo (neo-)adjuvant cancer treatment. Patients were excluded if they suffered from cognitive dysfunction (e.g. dementia or serious mental illness), physical impairments and/or other diseases (e.g. cardiovascular or lung diseases) that could affect their ability to perform physical activity and exercise. The studies were approved by the Regional Ethical Review Board in Uppsala (Dnr 2014/249, Dnr 2016/230/2), and all participants signed informed consent.

For the present study, we included those diagnosed with *breast cancer* with complete data at baseline, i.e. before starting (neo-)adjuvant cancer treatment. Data was pooled as the studies were similar in terms of inclusion criteria, context and assessment (timing and instruments) of the investigated variables.

### Measures

#### Sedentary time and physical activity

Sedentary time and physical activity were assessed with SenseWear Armband mini (SWA) at baseline. The SWA is a monitor combining a tri-axial accelerometer with heat/skin sensors, and has previously been validated in cancer survivors [19] and healthy adults [20, 21]. Participants were asked to wear the SWA 24 h a day for seven consecutive days. To reflect 1 week of sedentary time and physical activity, data from the SWA were included in the analyses if the SWA was worn for at least 4 days [22], including one weekend day [23] with a wear time of at least 80%/day [24].

The Professional 8.1 Software was used to provide SWA wear time and minutes spent in different levels of Metabolic Equivalent Task values (METs). Sedentary time, LPA and MVPA were determined using established cut-points of ≤ 1.5 METs [11], 1.6–2.9 METs and ≥ 3.0 METs, respectively [25]. Daily time spent sedentary, in LPA and MVPA, was calculated separately by summing minutes during waking hours for each valid day, where the criterion for the relevant intensity was met. Sedentary time, LPA and MVPA (expressed in min/day) were then converted to units of 30 min (e.g. 15 min = 0.5, 30 min = 1 and 60 min = 2) to facilitate the interpretation of the results.

#### Cancer-related fatigue

Cancer-related fatigue was assessed with the validated Multidimensional Fatigue Inventory questionnaire (MFI-20) [26] at baseline. The MFI is a 20-item scale designed to evaluate five dimensions of fatigue (subscales): *general fatigue* (i.e. general statements concerning an individual’s functioning), *physical fatigue* (i.e. physical sensation related to the feeling of tiredness), *mental fatigue* (i.e. cognitive symptoms related to fatigue), *reduced motivation* (i.e. lack of motivation to start any activity) and *reduced activity* (i.e. reduction in activity level) [27]. Participants were asked to indicate how true the 20 statements (items) had been for them over the last 7 days on a scale, ranging from 1 (“yes that is true”) to 5 (“no that is not true”). A score for each subscale was then calculated, ranging from 4 to 20 with higher scores indicating higher fatigue. Minimal clinically important change was defined as − 2 points per subscale [28].

#### Health-related quality of life

HRQoL was assessed with the subscale *Global health status/QoL* of the validated European Organisation for Research and Treatment of Cancer quality of life questionnaire (EORTC QLQ-C30) [29] at baseline. The subscale comprises two items. Participants were asked to rate their overall health and overall quality of life during the past week on a scale, ranging from 1 (Very poor) to 7 (Excellent). The raw scores were then transformed into a 0–100 scale in which higher scores indicate higher HRQoL. Minimal clinically important change was defined as + 10 points [30].

#### Covariates

Data were collected at baseline. Demographic data were self-reported and included age (years), education level (University vs. No university) and comorbidities (One or more comorbidities vs. No comorbidities). BMI (kg/m^2^) was calculated from measured height and weight by the study staff. Data about planned primary (neo-)adjuvant treatment were extracted from medical records. Due to the small proportion of participants planned to receive radiation or endocrine therapy as primary (neo-)adjuvant treatment, the variable was dichotomised as Chemotherapy vs. No chemotherapy.

### Statistical analysis

Descriptive characteristics are presented as mean and standard deviation (*SD*) for continuous variables and proportions as number (*n*) and percentage (%) for categorical variables.

Isotemporal substitution models were used to examine the effects of reallocating sedentary time to LPA or MVPA on cancer-related fatigue and HRQoL [31]. One model was run separately for each outcome. Each model included all exposures, except sedentary time, and a sum of time spent in all exposures (i.e. total time = sedentary + LPA + MVPA). Each model was adjusted for covariates: age, education level, comorbidities, BMI, planned primary (neo-)adjuvant treatment [32, 33] and study (Study 1 vs. Study 2 vs. Study 3). An example of the model is as follows: *HRQoL* = *LPA* + *MVPA* + *total time* + *covariates*. The coefficients can be interpreted as the mean change in the outcome when reallocating 30 min/day of sedentary time to 30 min/day of LPA or MVPA, whilst holding time spent in the other exposures constant.

To determine the daily amount of sedentary time needed to be reallocated to LPA or MVPA to produce minimal clinically important changes, sedentary time reallocated (30 min) were divided by the unstandardised regression coefficients and multiplied by the minimal clinically important change for each outcome.

The results are presented as unstandardised coefficients (*β*) with 95% confidence intervals (95% *CI*), and significance level set at 0.05. All statistical analyses were performed with SPSS (version 27).

## Results

### Participants

A total of 436 participants with breast cancer had complete data (accelerometer and questionnaires) at baseline and were therefore included in the present study. Participants’ characteristics are presented in Table 1. Participants’ mean age was 56 years (*SD* 11). The majority had undergone breast cancer surgery (88%), had a higher education (62%), at least one comorbidity (54%) and a normal BMI (50%). More than half of the participants were planned to receive chemotherapy as primary (neo-) adjuvant treatment (58%). Participants performed LPA and MVPA on average 254 (*SD* 81) and 71 (*SD* 48) min/day, respectively. They also spent on average 651 (*SD* 112) min/day sedentary (Table 1).

**Table 1:** Baseline characteristics of the study participants

	Total sample^a^ (*n* = 436)	Study 1 (*n* = 343)	Study 2 (*n* = 58)	Study 3 (*n* = 35)
Age, mean (*SD*)	56 (11)	56 (12)	57 (12)	53 (9)
High education (university), %	62	64	52	60
Living with partner, %	75	75	74	83
Occupation, %				
Working (part-time or full-time)	33	34	30	23
On sick leave	41	39	36	66
Retired	27	27	34	11
Comorbidities ≥ 1, %	54	56	50	37
Weight status, %				
Normal weight, BMI 18–24.9 kg/m^2^	50	51	43	48
Pre-obese, BMI 25–29.9 kg/m^2^	34	32	43	37
Obese, BMI > 29.9 kg/m^2^	16	17	14	14
Current exercise habits^b^, %				
Aerobic exercise since ≥ 6 months	39	34	62	47
Resistance exercise since ≥ 6 months	20	19	24	27
VO_2_max, mL/kg/min (*SD*)	30 (7)	30 (7)	29 (7)	29 (7)
Stage of breast cancer, %				
Stage 0 (in situ)	4	5	2	0
Stage 1 (T1N0M0)	52	54	65	21
Stage 2 (T1-2N1M0, T2-3N0M0)	39	38	31	59
Stage 3 (T1-2N2M0, T3N1-2M0)	5	4	0	21
Completed breast cancer surgery^c^, %	88	90	100	60
Planned primary (neo-)adjuvant treatment, %				
Chemotherapy	58	61	41	100
Radiation therapy	32	31	42	0
Endocrine therapy	9	8	18	0
MVPA, mean min/day (*SD*)	71 (48)	71 (48)	65 (35)	81 (58)
LPA, mean min/day (*SD*)	254 (81)	255 (84)	249 (81)	246 (56)
Sedentary time, mean min/day (*SD*)	651 (112)	649 (115)	664 (105)	645 (97)
Cancer-related fatigue (MFI, 4–20), mean (*SD*)^d^				
General fatigue	12 (4)	12 (4)	12 (4)	12 (4)
Physical fatigue	12 (4)	12 (4)	12 (4)	11 (4)
Reduced activity	11 (4)	11 (4)	11 (4)	10 (4)
Reduced motivation	9 (4)	9 (4)	9 (4)	9 (4)
Mental fatigue	10 (4)	10 (4)	10 (4)	10 (4)
HRQoL (EORTC QLQ-C30, 0–100), mean (*SD*)^e^	64 (19)	64 (20)	68 (19)	66 (19)

*BMI*: body mass index; *VO*_*2*_*max*: maximal volume of oxygen uptake; *MVPA*: moderate-to-vigorous intensity physical activity; *LPA*: light-intensity physical activity; *MFI*: Multidimensional Fatigue Inventory; *HRQoL*: health-related quality of life; *EORTC QLQ-C30*: European Organisation for Research and Treatment of Cancer

^a^Data from the Physical training and Cancer (Phys-Can) project, consisting of three studies: an RCT with a 2 × 2 factorial design (study 1), observational study (study 2) and a two-armed RCT (study 3)

^b^Assessed with Exercise Stage Assessment Instrument (5 categories, 1 = no intention to be physically active and 5 = physically active longer than 6 months)

^c^All participants planned for adjuvant treatment had undergone breast cancer surgery 4–6 weeks before the baseline measurements

^d^Higher scores indicate worse outcome

^e^Higher scores indicate better outcome

### Cancer-related fatigue

**Fig. 1:**
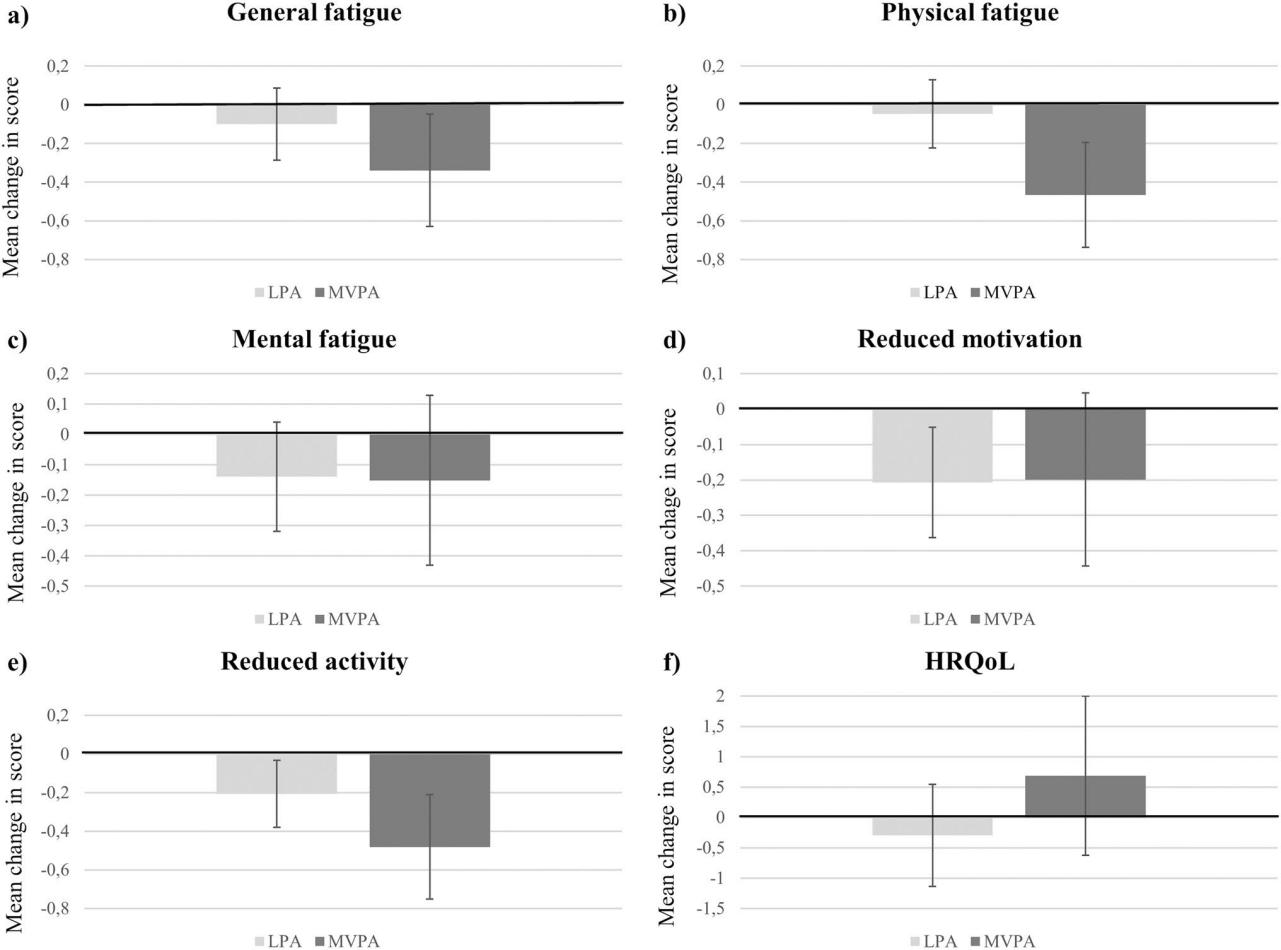
Effects of reallocating 30 min/day of sedentary time to light-intensity physical activity (LPA) or moderate-to-vigorous intensity physical activity (MVPA) on **a–e** different domains of cancer-related fatigue and **f** health-related quality of life (HRQoL), using isotemporal substitution modelling. Note that cancer-related fatigue was assessed with Multidimensional Fatigue Inventory (scale 4–20) and HRQoL with European Organisation for Research and Treatment of Cancer (scale 0–100). The vertical lines indicate the 95% confidence intervals. All models were adjusted for age, education level, comorbidities, body mass index, planned primary (neo-)adjuvant treatment and study

Results for the isotemporal substitution models are presented in Fig. 1a–e. Fatigue significantly decreased in two MFI dimensions when reallocating 30 min/day of sedentary time to LPA, i.e. *reduced motivation* (*β* =  − 0.21, 95% CI [− 0.36 to − 0.05], *p* = 0.008) and *reduced activity* (*β* =  − 0.21, 95% CI [− 0.38 to − 0.04], *p* = 0.018). Additionally, fatigue significantly decreased in three MFI dimensions when reallocating 30 min/day of sedentary time to MVPA, i.e. *general fatigue* (*β* =  − 0.34, 95% CI [− 0.63 to − 0.05], *p* = 0.020), *physical fatigue* (*β* =  − 0.47, 95% CI [− 0.74 to − 0.20], *p* = 0.001) and *reduced activity* (*β* =  − 0.48, 95% CI [− 0.75 to − 0.22], *p* < 0.001) (Fig. 1).

The amount of sedentary time needed to be reallocated to LPA to produce minimal clinically important changes (i.e. − 2 points) was 290 min/day for *reduced motivation* and 291 min/day for *reduced activity*. For MVPA, the corresponding amount was 177 min/day for *general fatigue*, 129 min/day for *physical fatigue* and 125 min/day for *reduced activity*.

### Health-related quality of life

No significant effects were observed on HRQoL when reallocating 30 min/day of sedentary time to LPA (*β* =  − 0.29, 95% CI [− 1.12 to 0.53], *p* = 0.486) or MVPA (*β* = 0.69, 95% CI [0.60 to 1.97], *p* = 0.295) (Fig. 1f). Due to the non-significant results, the daily amount of sedentary time needed to be reallocated to LPA or MVPA to produce minimal clinically important changes was not calculated for HRQoL.

## Discussion

The first aim of the present study was to examine how reallocation of sedentary time to LPA or MVPA affects fatigue and HRQoL in patients with breast cancer, using isotemporal substitution modelling. The second aim was to determine the daily amount of sedentary time needed to be reallocated to produce minimal clinically important changes in patients with breast cancer. Our results indicate that reallocating 30 min/day of sedentary time to LPA or MVPA decreased the level of cancer-related fatigue but did not affect HRQoL. To produce minimal clinically important changes in fatigue (i.e. − 2 points), the amount of sedentary time needed to be reallocated to LPA was ≈290 min/day and to MVPA was ≥ 125 min/day.

In accordance with our hypotheses, reallocating sedentary time to either LPA or MVPA was beneficial in terms of lower fatigue, with the reallocation to MVPA having the greatest effects. This is somewhat in line with the results from two previous studies, using isotemporal substitution with data from 463 kidney cancer survivors [34] and 149 non-Hodgkin lymphoma survivors [35]. In both studies, the authors found that reallocating sedentary time to MVPA was associated with lower fatigue, whilst one of the studies [34] also reported such effects when reallocating sedentary time to LPA. However, the amounts of time reallocation necessary to reach minimally clinical changes were lower compared to our findings: 65 min/day for LPA and 83 min/day for MVPA in the study of Tabaczynski et al. [34] and 30 min/day for MVPA in the study of Vallance et al. [35]. This discrepancy between the results from the above-mentioned studies and ours could be explained by the methodology used (fatigue was assessed with different questionnaires), the study samples having different cancer diagnoses and the timing of the assessments (prior to adjuvant treatment vs. post-treatment). Indeed, it is possible that the different questionnaires did not capture fatigue in the same manner or amplitude and that the amount of sedentary time reallocation necessary to reach minimally clinical changes differs across diagnosis and cancer phases. Another possible explanation is that our study sample had relatively low levels of fatigue at baseline, which may minimise the strength of the effects of reallocating sedentary time due to ceiling effects. Thus, it is possible that individuals with greater fatigue may experience clinical benefits with less daily sedentary time relocation to LPA or MVPA. Our results regarding the effect of reallocating sedentary time to LPA on cancer-related fatigue add to the growing evidence that LPA is beneficial for health amongst patients with cancer [8–10]. These results are of interest, especially for less physically active breast cancer populations as LPA may be a more feasible alternative than MVPA in this population. Contrary to our hypotheses, our findings suggest that the reallocation of sedentary time to LPA or MVPA does not have any effect on HRQoL. This is in contrast with findings from a previous study involving 753 patients with breast cancer [14], where reallocating 30 min of sedentary time to MVPA was associated with improved physical and functional well-being. Those divergent results could be explained by the use of different measurement instruments. Whilst in the present study, we used a subscale (Global health status/QoL of EORTC QLQ-C30) to assess HRQoL, Welch et al. [14] used another questionnaire (i.e. Functional Assessment of Cancer Treatment-Breast [FACT-B]) consisting of different domains that may have captured more variations in the outcome. However, our results are similar to the study of Vallance et al. [35], where no significant effects on HRQoL were found when reallocating 30 min of sedentary time to physical activity in non-Hodgkin lymphoma survivors. The present study provides evidence in support of an LPA and MVPA prescription for improving cancer-related fatigue in patients with breast cancer. Although a large amount of time reallocation is needed to produce clinically important changes in our study sample, our results indicate that (1) patients could be advised to replace sedentary time by either LPA or MVPA for improving cancer-related fatigue; (2) replacing sedentary time with LPA only, which may be a more feasible alternative in less physically active breast cancer populations, has a positive impact on cancer-related fatigue; and (3) for increased effects, an MVPA prescription is preferred.

This study has several methodological strengths, including a large sample and the objective measurement of sedentary time and physical activity via accelerometry. Additionally, the choice of analytical method (isotemporal substitution modelling) allowed us to simultaneously study how reallocating sedentary time to LPA or MVPA affected fatigue and HRQoL in patients with breast cancer. We chose to analyse bouts of 30-min reallocation to compare our results with other studies as the majority used this amount of time [14–16, 35]. It is also important to note that the length of the replaced bout (i.e. 30 min) does not affect the statistical significance of the results, only the strength of the associations. However, isotemporal substitution reflects the *theoretical* effects of reallocating one activity to another, rather than actual changes in behaviour. RCTs studying the effects of reducing actual sedentary time by increasing LPA or MVPA are therefore needed to confirm our results. This study also has limitations, including its cross-sectional design, limiting our ability to infer causality and the homogeneous study sample (i.e. mainly highly educated, physically active and relatively healthy women), restricting the generalisability of our results to broader cancer populations. It is also important to keep in mind that the outcome measures were collected prior to patients’ (neo-)adjuvant treatment, i.e. when cancer-related fatigue and HRQoL might be less affected compared to the time during and following (neo-)adjuvant treatment. Additionally, although we adjusted for potentially important covariates, our results may be subject to unmeasured confounding. For example, we did not have information on stage of cancer for all participants; however, we adjusted, instead, for planned primary (neo-)adjuvant treatment, which reflects the type of tumour.

## Conclusions

Our results suggest that reallocating sedentary time to LPA or MVPA has beneficial effects on cancer-related fatigue in patients with breast cancer, with MVPA having the greatest impact. However, a large amount of time reallocation is needed to produce clinically important changes in relatively healthy (e.g. low levels of fatigue) and physically active breast cancer populations. Future studies are therefore needed to evaluate such effects in broader cancer populations.

## Data Availability

The data that support the findings of this study are available upon reasonable request.
